# Western Range Limit, Population Density, and Flight Dynamics of the Fruit Pest *Grapholita inopinata* (Lepidoptera: Tortricidae) in Russia

**DOI:** 10.3390/life15040521

**Published:** 2025-03-22

**Authors:** Evgeny N. Akulov, Margarita G. Kovalenko, Julia A. Lovtsova, Dmitrii L. Musolin, Natalia I. Kirichenko

**Affiliations:** 1 Krasnoyarsk Branch of the All-Russian Plant Quarantine Center, Zhelyabova Str., 6/6, Krasnoyarsk 660020, Russia; akulich80@yandex.ru; 2 All-Russian Plant Quarantine Center, Bykovo, Moscow Oblast, Bykovo, Pogranichnaya str. 32, 140150, Russia; bush_zbs@mail.ru (M.G.K.); julialov@inbox.ru (J.A.L.); 3 European and Mediterranean Plant Protection Organization, 21 Boulevard Richard Lenoir, 75011 Paris, France; musolin@gmail.com; 4 Sukachev Institute of Forest, Siberian Branch of the Russian Academy of Sciences, Federal Research Center, Akademgorodok 50/28, Krasnoyarsk 660036, Russia; 5 Institute of Ecology and Geography, Siberian Federal University, Svobodny pr. 79, Krasnoyarsk 660041, Russia

**Keywords:** orchard pest, Manchurian fruit moth, modern range, population density, flight dynamics, European Russia

## Abstract

The Manchurian fruit moth, *Grapholita inopinata* (Heinrich) (Lepidoptera: Tortricidae), is an important pest of fruit crops, particularly apples (*Malus* spp., Rosaceae), and is classified as a quarantine pest in many European countries and other world regions. Until recently, this species was known only in Northeastern China, Japan, and Russia (from Eastern Siberia and the Far East). To determine the westernmost distribution of *G. inopinata* and assess its abundance, we conducted nine-year pheromone monitoring across 13 administrative regions of Russia from 2014 to 2018 and 2021 to 2024. A total of 1866 traps were deployed, capturing 31,962 *G. inopinata* specimens in 1811 traps. The species was newly detected in eight regions—seven in Asian Russia and one in European Russia (Perm Krai). These findings doubled the moth’s known range on the Asian continent and extended its western boundary to 56° E in European Russia. Between 2021 and 2024, *G. inopinata* was generally found at low densities across the surveyed regions (≤10 males per trap per week), with the exception of Perm Krai, Omsk, and Novosibirsk Oblasts, where moderate abundance (up to 38 males per trap per week) was recorded. In contrast, from 2014 to 2018, moderate to high population densities (up to 94 males per trap per week), including mass occurrences (over 100 males per trap per week), were observed in Krasnoyarsk Krai, with an absolute peak capture of 303 males in one trap in June 2017. Notably, in 2015–2017, male flight activity in southern Krasnoyarsk Krai exhibited two distinct peaks: one in mid-to-late June and another from late July to mid-August, indicating the development of two generations. This is the first-ever record of a bivoltine seasonal cycle for *G. inopinata* in Siberia. These findings are critical for improving pest risk assessments and developing early detection strategies, supporting more effective monitoring and management approaches of this orchard pest.

## 1. Introduction

The Manchurian fruit moth, *Grapholita inopinata* (Heinrich, 1928) (Lepidoptera: Tortricidae), is an East Palearctic pest of fruit crops, mostly apples (*Malus* spp., Rosaceae), but capable of attacking pears and other fruit crops from the subfamily Amygdaloideae [1,2,3,4]. Due to its potential to damage orchards and reduce fruit yields, *G. inopinata* is recognized as a threat to fruit production in non-native areas and has been listed as a quarantine species in some countries across both the Northern and Southern Hemispheres [5,6,7]. This species is of concern for Europe, where its regulation as a quarantine pest is recommended [7]. In 2019, *G. inopinata* was intercepted for the first time in Finland as an isolated finding in a pheromone trap, with no evidence of establishment of the species [8].

Historically, the moth’s range has been limited to East Asia, in particular, the eastern part of Asian Russia, northeastern China, and Japan [1,9,10]. In China, the species is widely distributed across the northeastern provinces [5]. In Japan, it is found on Honshu and Hokkaido [11,12]. The presence of the moth in Korea is debated. In the 1960s, larvae with chaetotaxy resembling *G. inopinata* were found in apples imported from Korea to Russia [9]. However, later studies found no evidence of the species presence in either South Korea [10] or North Korea [13].

In Russia, *G. inopinata* is naturally found in the Far East [1,2,14,15,16] and in some parts of Eastern Siberia [1,17]. In its native range, the pest can cause significant damage to fruit crops. For instance, in Eastern Siberia, damage from *G. inopinata* is known to reach 100% in some years [17,18], whereas in northeastern China, it has been reported to reach 50% of the total fruit loss in apples [19,20,21].

In the Russian Far East and Eastern Siberia, *G. inopinata* has one generation per year [1,15,22]. In China, it can have two generations, with adults flying in May–June and August–September [19,23]. Each generation can take up to two months [18,23]. Overlapping generations lead to larvae being present in fruits throughout the whole season [1].

Unlike some other moths with fruit-boring larvae, such as the oriental fruit moth *G. molesta* (Busck, 1916), *G. inopinata* adults are active during the day [1]. Females lay eggs on the surfaces of fruits [14,17] and occasionally on the underside of leaves [18]. A single female can produce up to 170 eggs [17,18]. Larvae begin feeding under the fruit’s skin, then move into the pulp and, eventually, feed on the seeds [17]. Typically, in one fruit, only one larva develops, rarely up to five larvae [24]. Late-instar larvae overwinter in silk cocoons in bark cavities, in soil, or among fallen leaves [3,15,18]. Overwintering in fruit crates is also possible [15]. Larvae are cold resistant [17] and pupate in late spring [1,14,17].

One of the efficient approaches to detect the presence of fruit-boring moths, even at their low population density, is the use of pheromone sticky traps [25,26,27,28,29]. Such traps capture adult moths, providing valuable data for early detection and monitoring. Synthetic pheromones which are used in traps cannot always be highly species-specific, as they are often composed of components shared by closely related species [29,30,31,32]. For instance, the synthetic pheromone of *G. molesta* shares some components with those of other fruit borers, which can lead to cross-attraction and complicate pest management [33,34,35]. Such non-specificity can, however, serve as an advantage, expanding its potential applications for broader ecological monitoring or for pest control [36]. The same pheromones that attract the target species can also attract closely related species, providing insights into the population dynamics of several pests simultaneously [37,38,39,40]. This was observed in different fruit pests, when pheromone traps designed for one pest species inadvertently captured related species, enabling their monitoring or control [36,41].

The pheromone monitoring of *G. molesta*, a quarantine pest in Russia, revealed that other moths from the family Tortricidae, including *G. inopinata*, were attracted to the traps [22,39]. This led to the detection of *G. inopinata* in southern Siberia in 2010–2013. Its presence in Western Siberia and further west, in European Russia, remains uncertain [22].

For nine years (2014–2018 and 2021–2024), we performed pheromone monitoring in 13 regions of Russia, focusing on Western Siberia and the European part of the country in order to (1) detect the presence of *G. inopinata* in the regions where it had not been documented yet, and thus to clarify the western limit of its range, (2) assess its abundance, (3) study the species flight seasonal dynamics, and define the number of generations the pest can annually produce. Here, we present the results of the study and highlight the importance of pheromone trapping, addressing various questions linked to the pest distribution and population dynamics, which are the basis for defining effective pest management strategies.

## 2. Materials and Methods

### 2.1. Study Region

**Figure 1:**
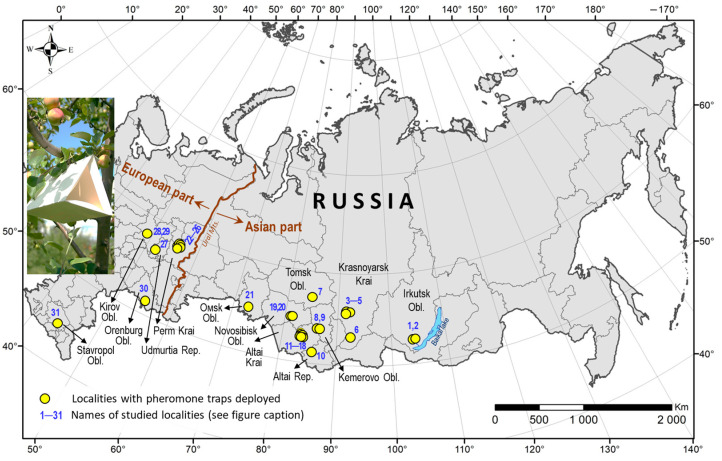
The administrative regions and localities in Russia, where pheromone monitoring was performed. Localities: private gardens (1, 2, 4, 5, 7–11, 13–19, and 21–31), state-owned gardens—V.M. Krutovsky Botanical Garden (3), Minusinsk Experimental Station for Horticulture and Melon Growing of the Krasnoyarsk Research Institute of Agriculture (6), Central Siberian Botanical Garden of the Siberian Branch of the Russian Academy of Sciences (20), and planted apple trees as ornamentals in a periurban area (12). The full names of the localities are given in Table S1. The map was produced using ArcGIS 10.6.1 software [42]. The photo of a pheromone trap on an apple tree in the insertion: E.N. Akulov.

The study was conducted from May to October in 2014–2018 and 2021–2024 in 31 localities of 13 administrative regions of Russia (Figure 1, Table S1).

In the Asian part of Russia, observations were made in eight regions: Irkutsk Oblast (2 localities), Krasnoyarsk Krai (4), Tomsk Oblast (1), Kemerovo Oblast (2), Altai Krai (8), Altai Republic (1), Novosibirsk Oblast (2), and Omsk Oblast (1). In the European part of Russia, the research encompassed five regions: Perm Krai (5 localities), Udmurtia (1), Kirov Oblast (2), Orenburg Oblast (1), and Stavropol Krai (1). Surveys were conducted in 3 governmental gardens, 27 private gardens, and on 1 ornamental plot. These gardens were located in the suburban zones of the major cities of these administrative regions or in the settlements or villages of the region.

**Figure 2:**
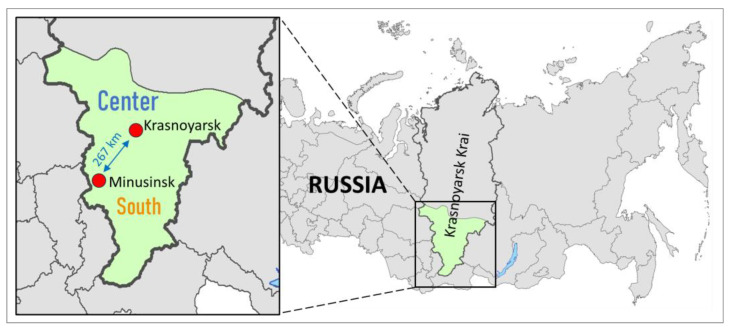
The area in Krasnoyarsk Krai, where regular pheromone monitoring was performed in 2014–2018 to access the flight dynamics of *Grapholita inopinata*. The map was produced using ArcGIS 10.6.1 software [42].

In the aforementioned regions (excluding Krasnoyarsk Krai), opportunistic field studies were carried out for 4 years (2021–2024) to reveal the presence of *G. inopinata* and gain insights into its abundance. In Krasnoyarsk Krai, where *G. inopinata* was first detected in 2011 [22], extensive field work was performed for 5 years (2014–2018) in the central part of the region (Krasnoyarsk city, 2014–2018) and in the southern part (near Minusinsk city, 2015–2018; Figure 2). Data from Krasnoyarsk and Minusinsk were used to check the abundance and explore the seasonal flight dynamics of *G. inopinata* based on the number of individuals captured in pheromone traps.

Krasnoyarsk and Minusinsk are the main localities of Krasnoyarsk Krai where fruit crops such as pome fruits (apple and pear) and stone fruits (plum, cherry, and apricot) are intensively cultivated in both state-owned and private orchards. These areas, particularly Krasnoyarsk, also serve as key hubs for the transport, storage, and trade of fruits imported from Central and East Asia, regions known for hosting a variety of fruit-boring insect species.

Krasnoyarsk is located in a basin formed by the northernmost spurs of the Eastern Sayan Mountains and is characterized by a continental climate with sharply fluctuating temperatures. Winters are long and cold, with a daily average temperature of −16 °C, an average minimum of −19.2 °C, and an absolute minimum of −52.8 °C in January. Summers are short and warm, with a daily average of +19.1 °C, an average maximum of +23.5 °C, and an absolute maximum of +37.2 °C in July. The annual precipitation is about 507 mm [43,44].

The Minusinsk Basin, a predominantly flat area with fertile soil, is bordered by the Kuznetsk Alatau to the west and the Sayan Mountains to the east. The winters are cold (daily average −17.6 °C, average minimum −23.1 °C, and absolute minimum −52.2 °C in January), summers are hot (daily average +20.0 °C, average maximum +27.1 °C, and absolute maximum +39.3 °C in July), with abundant sunny days [44], and an annual precipitation of 534 mm [45].

### 2.2. Pheromone Monitoring

**Figure 3:**
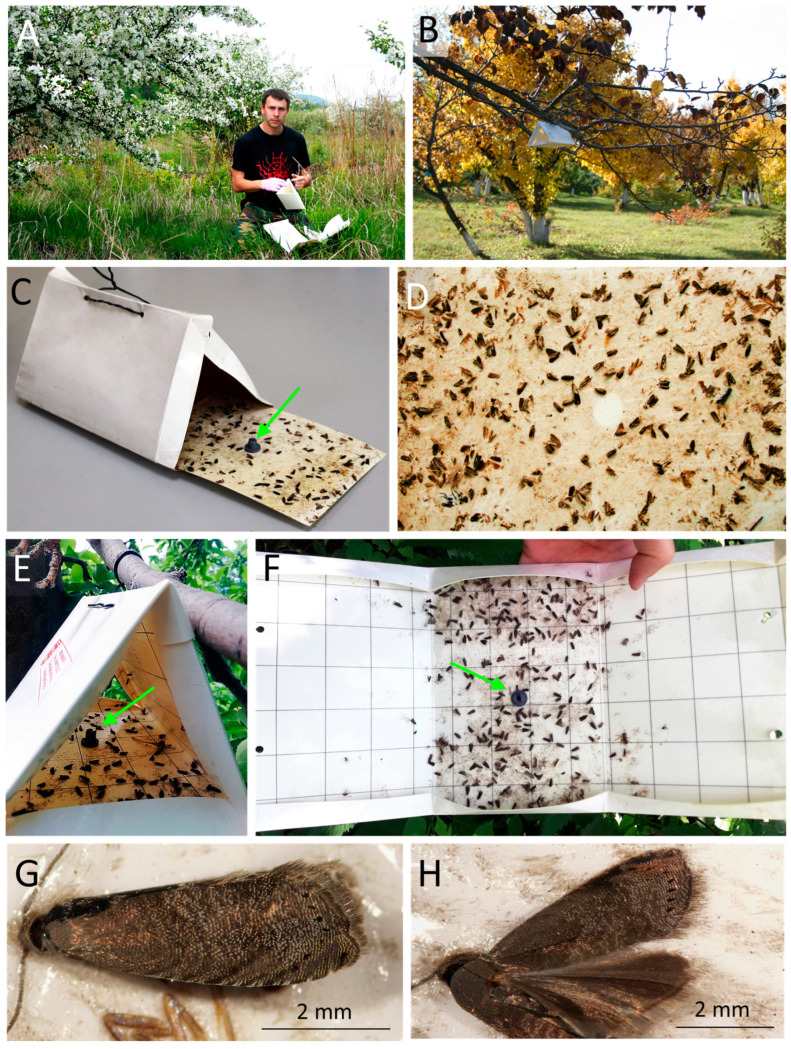
Pheromone monitoring in an orchard of the V.M. Krutovsky Botanical Garden, Krasnoyarsk city, Krasnoyarsk Krai, Russia, June–September 2018 (**A**–**D**,**G**,**H**) and Altai Krai, July 2022 (**E**,**F**). (**A**,**B**)—deploying the traps in the garden; (**C**,**D**)—early version of the trap with and an insert; close-up of the insert with glued moths, with the majority being *G. inopinata*; (**E**)—the trap with the sticky sides and the pheromone dispenser (indicated by the green arrow) and captured insects; (**F**)—an opened pheromone trap with numerous moths inside; (**G**,**H**)—*G. inopinata* adults trapped in the glue on the sticky surface. Photos: E.N. Akulov. Photo A is published with the permission of the photographed co-author, E.N. Akulov.

For pheromone monitoring aimed at detecting *G. inopinata*, delta-shaped pheromone traps (19 × 13 × 11 cm) produced from laminated paper by the All-Russian Plant Quarantine Center “VNIIKR” (Bykovo, Moscow Oblast) were used (Figure 3A–D). Inside each trap, the laminated paper insert (18.5 × 12.5 cm with 2 × 2 cm grids) was placed at the bottom, with Polifix glue (TU 2387-002-5584-1212-2002) (VNIIKR, Bykovo, Moscow Oblast) applied evenly to its surface, allowing moths to easily attach to it (Figure 3C,D).

The traps of this type (i.e., featuring removable inserts with an adhesive surface) were deployed in Krasnoyarsk Krai in 2014–2018. Improved pheromone traps produced by the same organization were used in all other administrative regions in 2021–2024. The improved traps had the same shape and size as the earlier version, but the glue was spread on all the internal walls of these traps (Figure 3E,F). For clarity, we use the term ’trap’ instead of ’insert’ when referring to data from old-type traps. In the modern practice, traps are entirely replaced each time, as the adhesive substance (glue) is applied directly to the inner walls, making each trap single use. In contrast, the old-type pheromone traps were reusable, with adhesive inserts regularly replaced. Thus, each ’insert’ effectively functioned as a separate trap.

A synthetic sex pheromone of the oriental codling moth *G. molesta* (composed of Z8-dodecenyl acetate, E8-dodecenyl acetate, and Z8-dodecenol) [46] was used in our study. Based on our earlier field observations, this pheromone composition effectively attracted not only *G. molesta* but also *G. inopinata* [39]. A round-shaped rubber dispenser (10 mm in diameter and 10 mm in height) containing the synthetic sex pheromone of *G. molesta* was placed at the center of the insert inside the traps. In the new trap design (with glue applied to the bottom), the dispenser was placed at the center of the inner surface of the trap (Figure 3C,E,F).

Overall, 1866 traps were used during nine-year monitoring in all the studied regions of Russia. In each region, up to 5 traps at once were placed in 1–8 localities, with up to 25 traps used in a few cases. In 12 administrative regions (except Krasnoyarsk Krai), the traps were deployed from the beginning of June to mid-July, a period during which, according to our earlier observations, there was a high likelihood of detecting *G. inopinata* adults [39]. In these regions, the traps remained in place for about a month without being replaced. At the end of the exposure period, the traps were carefully removed and packed to ensure that the adhesive surface and the moths collected on it did not come into contact with the other sides of the traps. The traps were transported to the VNIIKR Laboratory in Krasnoyarsk for the investigation of the captures and species identification.

In Krasnoyarsk Krai (in its central and southern parts), routine monitoring was performed, with up to 9 traps deployed at the start of monitoring in the botanical gardens or private orchards from the end of May to October in 2014–2018. In this region, insects captured in the pheromone traps were collected every week. For that, the inserts with glued adult moths were removed from the traps, and a new insert with glue was placed in each trap (in this case, replaced inserts are considered in our work as individual traps, as described above). The sampling of catches (with trap replacement to a new one) was conducted every week, up to 20 times per season. The dispenser was transferred from the used insert to the new one but was not used for longer than 40 days according to the producer’s recommendations [46].

### 2.3. Species Identification

*Grapholita inopinata* moths captured in pheromone traps often had forewings abraded due to attachment to the sticky surface of the pheromone traps. In exceptional cases, when the moths became attached by the inner side of their forewings to the sticky surface of the traps, the forewing pattern was preserved (Figure 3G,H), and the provisional identification was possible when sorting the moths caught in the pheromone traps.

*Grapholita inopinata* can be easily distinguished from similar species based on the male genital morphology. Thus, in our study, the identification of all captured individuals of *G. inopinata* was based on the structure of the male genitalia [1].

To process a large number of specimens, a noninvasive approach was used. In fresh adult specimens, gentle pressure was applied to the penultimate and ultimate abdominal segments to induce the prolapse of genital valvae for identification. In dry specimens and in problematic cases, genitalia were dissected from the insects’ abdomens using the standard method [47], with our adjustment (heating the abdomens in 20% KOH solution at 70 °C for 10 min). Male genitalia from regions other than Krasnoyarsk Krai were all analyzed through dissection. In total, the male genitalia of 31,692 *G. inopinata* specimens were examined. The genitalia from the majority of the specimens were kept in 95% glycerol or in 95% ethanol in microvials (1.5 mL) with hermetic lids. The male genitalia from 40 specimens were embedded in Canada balsam as permanent slides. All materials are stored in the VNIIKR Laboratory, Krasnoyarsk.

### 2.4. Assesment of G. inopinata Abundance

The data on *G. inopinata* catches from the localities within one administrative region were analyzed together as these localities were situated not far away from each other (within 20 km diameter), and no significant difference between the moth’s catches was detected. In Krasnoyarsk Krai, estimations were made separately for the central part of the region and the southern part (Figure 1 and Figure 2).

The total number of *G. inopinata* catches per administrative region per year was estimated. For each administrative region, the number of *G. inopinata* adults in the pheromone traps (or the inserts in Krasnoyarsk Krai) was calculated for each sampling date. Since in most administrative regions, monitoring was performed more or less for one month (roughly from mid- or end of June to mid- or end of July, covering the flight peak(s)), and the data on *G. inopinata* catches were calculated for this period (per trap per week), including for Krasnoyarsk Krai (where monitoring was performed for four months). Such adjustment allowed the comparison of abundance across all regions and years. As no universally standardized ranking system for the abundance of *Grapholita* spp. captured in pheromone traps exists, we categorized moth abundance into qualitative levels based on our experience: low (≤10 individuals per trap per week), moderate (11–50), high (51–100), and mass abundance (>100). The Mann–Whitney U-test was used to compare relative abundance between the regions in the same year.

### 2.5. Assessment of G. inopinata Flight Dynamics

This analysis was conducted exclusively for Krasnoyarsk Krai, where comprehensive pheromone monitoring was carried out in 2014–2018 (see paragraph 2.2). Data on moths trapped in the central part of the region (2014–2018) and the southern part (2015–2018) were analyzed separately. The number of *G. inopinata* individuals was averaged per trap for the sampling date in each season. The mean data on the moth catches (± standard error) were plotted together with average daily temperature data. Average daily temperatures for the central and southern parts of the region were analyzed using weather records from Krasnoyarsk and Minusinsk [48,49] for the studied period (May–October) for 2014–2018.

The flight activity of *G. inopinata* males and the mean daily temperature dynamics were approximated by a polynomial function of the sixth and second order, respectively, in Statistica 14.0 (TIBCO Software Inc., Palo Alto, CA, USA).

### 2.6. Photographing and Mapping

Adult specimens were photographed with a Canon EOS 6D digital SLR camera (Canon, Tokyo, Japan) with a Canon EF 100 mm f/2.8 L IS USM macro lens, which was fixed on a Manfrotto tripod with a smooth motion along the axis of the optical system. A Canon Macro Ring Lite ring flash (Canon, Tokyo, Japan) was used to evenly illuminate the specimens without glare. Male genitalia were photographed using an Olympus CX 41 microscope (Olympus Corporation, Tokyo, Japan). The photographs were edited in Adobe Photoshop v. 24.0.1 (Adobe, San Jose, CA, USA). For mapping the sampled localities and *G. inopinata* range, ArcGIS 10.6.1 software (ESRI, Redlands, CA, USA) was used [42].

## 3. Results

### 3.1. Novel Regional Records of G. inopinata

*Materials examined:* Russia: **Kemerovo Oblast**, Kuzbass, Kemero district, Urmanay vil., private garden, 55.2854° N, 86.3745° E, 164 m a.s.l., 17.VI–03.VII.2021, 6 males; Mariinsk district, Mariinsk, private garden, 55.2854° N, 86.3745°E, 134 m a.s.l., 03–18.VII.2021, 10 males, A. Otteva coll. (a new record). **Tomsk Oblast**, Tomsk district, Belousovo vil., private garden, 56.3063° N, 85.1833° E, 171 m a.s.l., 15.VI–04.VII.2021, 1 male, D. Kuleshov coll. (a new record). **Novosibirsk Oblast**, Novosibirsk district, Krasnoobsk, private garden, 54.9353° N, 83.0283° E, 99 m a.s.l., 15–25.VI.2021, 14 males; Novosibirsk, Botanical Garden of the Siberian Branch of the Russian Academy of Sciences, 54.8208° N, 83.1058° E, 158 m a.s.l., 15–25.VI.2021, 7 males, M. Tomoshevich coll. (a new record). **Altai Krai**, Slavgorod, 52.9971° N, 78.6487° E, 117 m a.s.l., 18–28.VI.2022, 61 males; Kulunda vil., ornamental planting of apple trees, 52.5638° N, 78.9465° E, 139 m a.s.l., 18–28.VI.2021 and 11–21.VII.2022, 123 males; Zavyalovsky district, Glubokoe vil., private garden, 52.9902° N, 80.6840° E, 162 m a.s.l., 11–26.VI.2022, 39 males; Rubtsovsky district, Veseloyarsk settl., private garden, 51.2829° N, 81.1081° E, 232 m a.s.l., 11–26.VI.2022, 1 male; Loktevsky district, Ustyanka settl., the community of private gardens «Shakhter 2», 51.1439° N, 81.5947° E, 271 m a.s.l., 15–25.VII.2022, 47 males; Georgievka settl., the community of private gardens «Shakhter 1», 51.1397° N, 81.5077° E, 262 m a.s.l., 15–25.VII.2022, 74 males; Zmeinogorsk district, Zmeinogorsk, private garden, 51.1539° N, 82.1985° E, 369 m a.s.l., 11–21.VI.2022, 64 males; Barnaul, private garden, 53.3468° N, 83.7769° E, 230 m a.s.l., 11.VI–25.VII.2022, 126 males, L. Snigireva coll. (a new record). **Altai Republic**, Gorno-Altaysk, private garden, 51.9578° N, 85.9606° E, 302 m a.s.l., 11–26.VI.2022, 50 males, L. Snigireva coll. (a new record). **Omsk Oblast**, Omsk, the communities of private gardens «Podrost» and «Teplichniy 2», 54.8840° N, 73.3339° E, 75 m a.s.l., 10.VI–13.VII.2022, 293 males, A. Terebilov coll. (a new record). **Perm Krai**, Perm district, Kondratovo vil., private garden, 57.9759° N, 56.1066° E, 97 m a.s.l., 22.VI–07.VII.2023, 83 males, I. Sokolova coll. (a new record). **Irkutsk Oblast**, Irkutsk, Selivanikha settl., 52.2997° N, 104.2354° E, 429 m a.s.l., 14.VI–04.VII.2024, 45 males; ibidem Shelekhovsky district, garden society «Shirokaya pad’», 52.1372° N, 104.0611° E, 484 m a.s.l., 10.VI.2021, 47 males, staff of the VNIIKR (Irkutsk Branch) coll. (a new record). In all cases, the specimens of *G. inopinata* were captured in the pheromone traps; E.N. Akulov det.

*Additional specimens examined:* Russia: **Krasnoyarsk Krai**, Krasnoyarsk, Sverdlovsky district, V.M. Krutovsky Botanical Garden, 55.9711° N, 92.7487°, 45 m a.s.l., Studgorodok district, the community of private gardens «Pobeda», 56.0052° N, 92.7862° E, 296 m a.s.l., Vetluzhanka district, the community of private gardens «KZK-2», 56.0345° N, 92.7394°, 240 m a.s.l., 17.VI–23.IX.2014, 714 males of *G. inopinata* in total in above listed localities; ibidem, 04.VI–01.X.2015, 1493 males; ibidem, 01.VI–28.IX.2016, 3068 males; ibidem, 17.V–27.IX.2017, 4499 males; ibidem, 30.V–26.IX.2018, 1767 males, E.N. Akulov coll.; ibidem, Minusinsk district, Opytnoe pole settl., Minusinsk Experimental Station for Horticulture and Melon Growing of the Krasnoyarsk Research Institute of Agriculture, 53.6748° N, 91.6715° E, 255 m a.s.l., 04.VI–17.IX.2015, 1245 males; ibidem, 25.V–14.IX.2016, 2455 males; ibidem 25.V–13.IX.2017, 8944 males, ibidem 23.V–12.IX.2018, 6416 males, A. Medvedenko coll. In all cases, the specimens of *G. inopinata* were captured in the pheromone traps; E.N. Akulov det.

**Figure 4:**
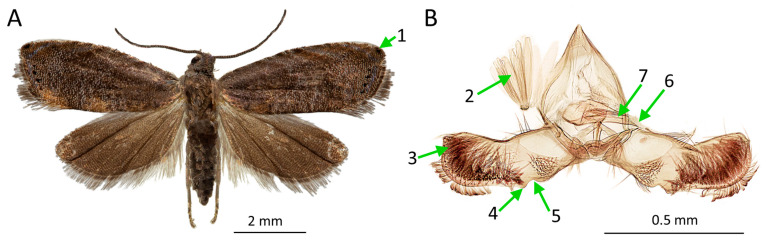
Male of *Grapholita inopinata*, Krasnoyarsk, 21 June 2022 (**A**), and Perm Oblast, 22 July 2023 (**B**). (**A**)—spread adult specimen; (**B**)—genitalia with the main parts and diagnostic characters indicated; 1—black dot at the apex of the forewing; 2—scales of the coremata; 3—cucullus; 4—small projection of the ventral base of the cucullus; 5—slight notch, separating the cucullus from the main part of the valvae; 6—aedeagus; 7—cornuti. Photos: E.N. Akulov, J.A. Lovtsova, and M.G. Kovalenko.

*Adult morphology:* Morphologically, all specimens of *G. inopinata* collected in Siberia and the European part of Russia (i.e., 31,962 specimens) aligned with the descriptions provided in the key [1]. The wingspan of the studied specimens ranged from 10 to 12 mm. The forewing pattern was weakly developed, with a grayish–brown background (Figure 4A). In well-preserved specimens, the outer edge of the forewings exhibited an indistinct speculum, with four (occasionally three) black dots and the characteristic black dot at the apex of the forewing (Figure 4A). In older moths, particularly those with worn wings, and in most specimens captured on the sticky surface of the pheromone traps, the forewing pattern was significantly abraded.

In all the analyzed male specimens, including those from Perm Oblast (the European part of Russia), the valva was wide and curved at the middle (Figure 4B). The cucullus was roughly the same width as the main part of the valva and separated by a slight notch (Figure 4B). In contrast, the cucullus of other *Grapholita* species (e.g., *G. molesta* and *G. funebrana* (Treitschke, 1835)) is clearly wider than the main part of the valva and are separated by a transverse fold. The ventral base of the cucullus bore a small projection. The aedeagus was nearly straight, distinctly narrowing toward the apex, and featured several long cornuti. In some specimens, cornuti were absent, indicating that these males had already mated and transferred their cornuti to the females. The aedeagus without cornuti has been reported in some taxonomic and faunistic publications [1,4]. In the image of the aedeagus in the original description [50], the cornuti are present. The scales of the coremata were lanceolate in shape.

**Figure 5:**
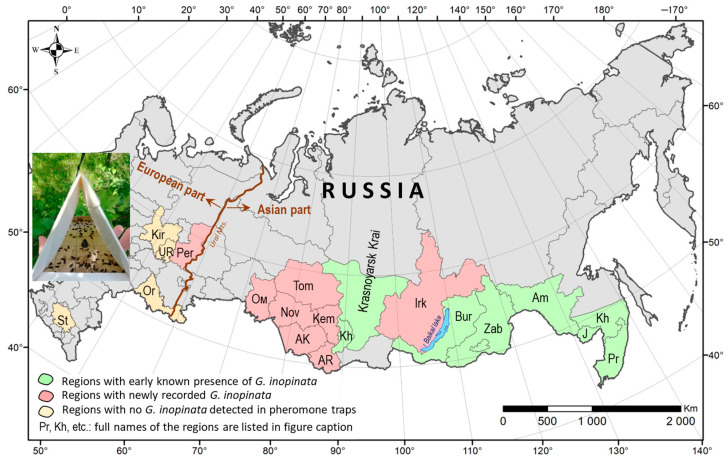
Novel regional records of *Grapholita inopinata* in the Asian and European parts of Russia in 2014–2024. Regions (form east to west): Pr—Primorsky Krai, J—Jewish Autonomous Oblast, Kh—Khabarovsk Krai, Am—Amur Oblast, Zab—Zabaykalsky Krai, Bur—Buryatia Republic, Irk—Irkutsk Oblast, Kh—Khakassia Republic, AR—Altai Republic, AK—Altai Krai, Kem—Kemerovo Oblast, Tom—Tomsk Oblast, Nov—Novosibirsk Oblast, Om—Omsk Oblast, Per—Perm Krai, UR—Udmurtia Republic, Kir—Kirov Oblast, Or—Orenburg Oblast, and St—Stavropol Krai. In the insertion, the triangular pheromone trap on an apple tree is shown. The map was produced using ArcGIS 10.6.1 software [42]. The photo of a pheromone trap with captured moths, in the insertion: E.N. Akulov.

*Novel regional records: Grapholita inopinata* was captured in the pheromone traps in 8 out of 13 administrative regions of Russia included in the study (62%). In 7 of these regions, the moth was recorded for the first time. Six of these regions are in Siberia—Kemerovo, Tomsk, Novosibirsk Oblasts (2021), Altai Republic (2022), Altai Krai (2022), Omsk Oblast (2022), and Irkutsk Oblast (2024)—and one is in the European part of Russia, Perm Krai (2023) (Figure 5). In Krasnoyarsk Krai, where the moth was first documented by our team in 2010 [22], it was repeatedly observed in all surveyed years (2014–2018). During 2021–2024, *G. inopinata* was not detected in Udmurtia Republic, Kirov Oblast, Orenburg Oblast, and Stavropol Krai in the European part of Russia (Figure 5). Two of them (namely, Udmurtia and Kirov Oblast) border Perm Krai, where *G. inopinata* was revealed.

Overall, our new records doubled the number of regions where *G. inopinata* is present in Russia, increasing it from 8, where the moth was already known (Primorsky Krai, Khabarosk Krai, Jewish Autonomous Oblast, Amur Oblast, Zabaykalsky Krai, Krasnoyarsk Krai, and Khakassia Republic) to 16 (Figure 5). Additionally, they extended the western boundary of its distribution from 91° E to 56° E longitude, i.e., by 35° of longitude.

### 3.2. Abundance of G. inopinata

A total of 31,692 *G. inopinata* males were captured in pheromone traps in 1811 traps across nine studied regions of Russia over a 9-year period (2014–2018 and 2021–2024) (Table S1). The majority—30,601 specimens (97%)—were collected in Krasnoyarsk Krai, where intensive monitoring was conducted from 2014 to 2018. The remaining 1091 specimens (3%) were recorded in eight other regions: in seven in the Asian part of Russia (Irkutsk, Kemerovo, Tomsk, Novosibirsk, Omsk Oblasts, Altai Krai, and the Altai Republic) and in one in the European part of Russia (Perm Krai).

**Figure 6:**
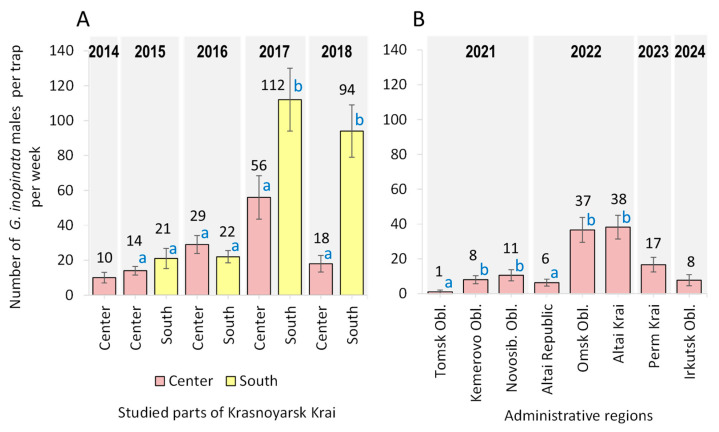
Abundance of *Grapholita inopinata* in nine Russian regions over one month (mid-June to mid-July) in different years: (**A**) Krasnoyarsk Krai and (**B**) eight other regions. Bars marked with different letters indicate significant differences between regions within the same year (Mann–Whitney U-test, *p* < 0.05). No statistical comparisons were made between regions surveyed in different years, as variations in environmental conditions, sampling efforts, and other uncontrolled factors could influence the results, making comparisons unreliable.

The relative abundance of *G. inopinata* (per trap per week) varied significantly across regions. In Krasnoyarsk Krai, the most extensively studied region, where central and southern areas were analyzed separately, the lowest abundance (10 moths per trap per week) was recorded in the central part, while the highest, corresponding to a mass abundance rate (112 moths per trap per week) was observed in the south (Figure 6A). In 2014–2016, no significant difference was detected in *G. inopinata* abundance between the central and southern parts of Krasnoyarsk Krai. However, in 2017 and 2018, moth densities increased notably in the south (up to outbreaking levels), whereas populations in the central region remained significantly lower—twice lower in 2017 and five times lower in 2018 compared to the south in the same years (Figure 6A). The maximum capture was 303 *G. inopinata* males per week in a single trap in the southern part of the region on 28 July 2017.

In the eight other regions of Russia, where trapping was less intensive, *G. inopinata* was recorded at low densities—fewer than 10 specimens per trap per week—in Tomsk, Kemerovo, and Irkutsk Oblasts, as well as in the Altai Republic (Figure 6B). In contrast, in Novosibirsk, Omsk, and Altai Krai, as well as Perm Krai, the species was present at a moderate density, ranging 11–50 individuals per trap per week (Figure 6B).

In the Asian part of Russia, within regions where *G. inopinata* was first recorded between 2021 and 2024, the highest weekly capture on a single trap was 72 individuals, observed in Altai Krai on 28 June 2022. In the European part of Russia, specifically in Perm Krai, the highest recorded number of moths in one trap within one week was 37 individuals, as documented on 22 June 2023.

### 3.3. Flight Dynamics of G. inopinata

In 2014–2018, in the central part of Krasnoyarsk Krai, the first *G. inopinata* males for each year were detected in pheromone traps from 4 June to 26 September. In the southern part of the region, males typically appeared about two weeks earlier, starting around 23 May, with the latest captures recorded by 20 September. In both regions, *G. inopinata* males were continuously trapped for nearly four months, with no significant difference in the duration of their occurrence: 105 ± 3.8 days in the center vs. 112 ± 4.5 days in the south (Mann–Whitney U-test: U = 3.5, Z =−1.47, N_center_ = 75, N_south_ = 36, and *p* = 0.14).

**Figure 7:**
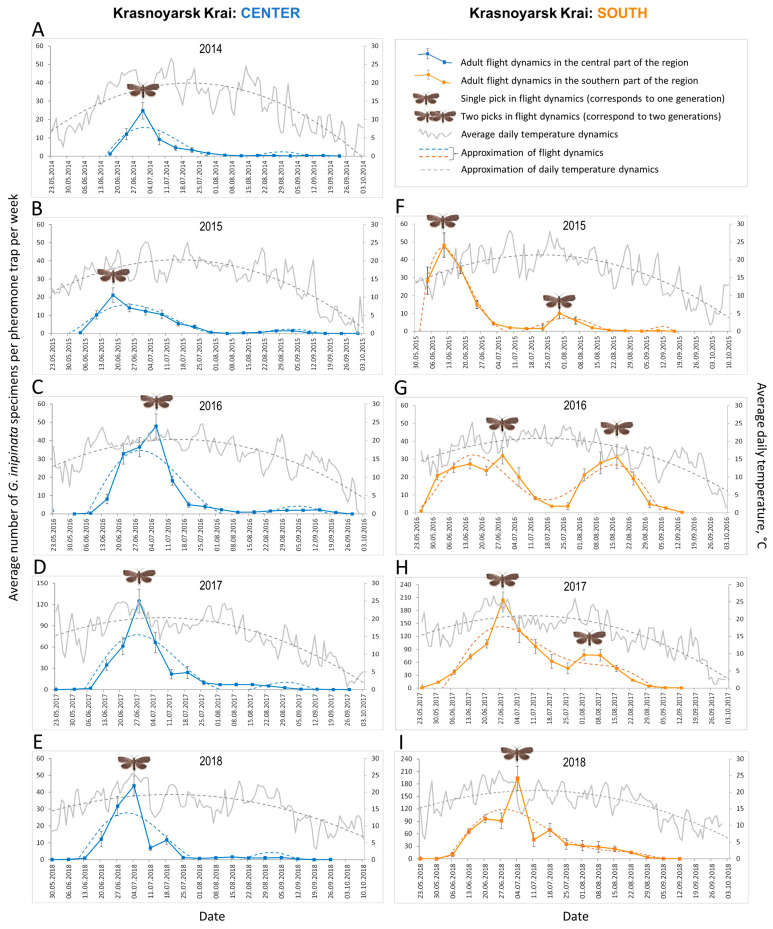
Flight dynamics of *Grapholita inopinata* males in the central (**A–E**) and southern (**F–I**) parts of Krasnoyarsk Krai (2014–2018), based on weekly records over a period of four months each year, and the dynamics of daily temperature. The presence of one peak means the development of one annual generation, two peaks–two annual generations. Flight dynamics of the moth males and mean daily temperature trends were approximated using a polynomial function (for the statistics, see Table S2).

In the central part of the region, a single peak in *G. inopinata* flight dynamics was documented each year over the 5-year study period (Figure 7A–E). This peak occurred between 19 June (the earliest, in 2015) and 6 July (the latest, in 2016) (Figure 7B,C).

In the southern region, both single- and double-peak patterns of *G. inopinata* flight dynamics were recorded, depending on the year and seasonal temperature fluctuations (Figure 7F–I). A single peak, indicating the presence of one generation per year, was observed in 2018 (Figure 7I). In contrast, two peaks were recorded in 2015–2017 (Figure 7F–H). The first peak occurred as early as 11 June (2015) and as late as 29 June (2016), while the second peak appeared as early as 30 July (2015) and as late as 17 August (2016) (Figure 7F,G). In 2017, peaks were recorded on 28 June (the first peak) and 9 August (the second peak), respectively (Figure 7H). The interval between the two peaks averaged 48 ± 2.3 days, reflecting the time required for the development of the second generation.

After completing two generations per year in 2015 and 2016 in the southern region, *G. inopinata* males were captured in mass in the following two years (2017–2018). During the first peak, the weekly catch averaged 200 ± 19 males, with a maximum weekly capture of 303 males in a single trap recorded on 28 June 2016, and 254 males on 4 July 2017.

In both the central and southern parts of Krasnoyarsk Krai, flight dynamics were effectively modeled using a sixth-degree polynomial (Figure 7, Table S2(A)). The polynomial regression explained a significant portion of the observed variability and closely fit the moth seasonal dynamics, particularly the two-peak pattern (Figure 7F–H).

During the study period (late May to early October), average daily temperatures fluctuated notably. Their dynamics were effectively approximated using a quadratic function (Figure 7, Table S2(B)). In both the central and southern parts of the region, the first peak in moth flight coincided with the average daily temperature of +19 to +25 °C. In the south, the second peak occurred when the average daily temperatures ranged from +18 to +24 °C.

## 4. Discussion

**Figure 8:**
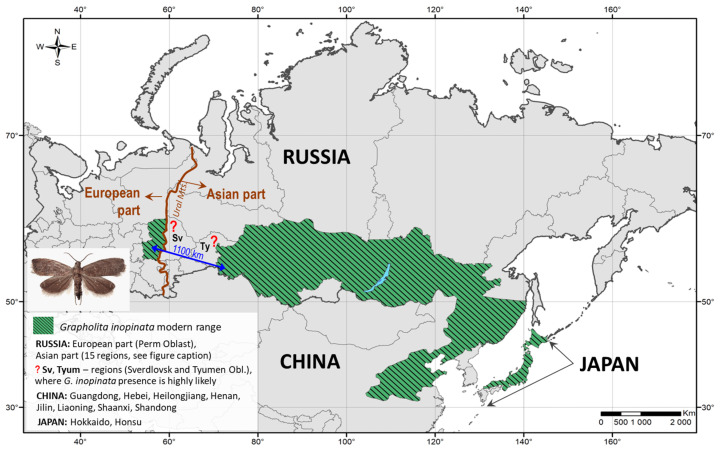
Modern range of *Grapholita inopinata* with the westernmost distribution boundary in the European part of Russia (Perm Oblast, the region preceding the Ural Mountains). Sv—Sverdlovsk Oblast, Ty—Tyumen Oblast. The distance of 1100 km separates the two closest locations: one in the west (in Perm Oblast) and the other in the east (in Omsk Oblast), where *G. inopinata* was documented in the present study. The data on China and Japan extracted from the EPPO Global Database [7]. The map was produced using ArcGIS 10.6.1 software [42]. The photo of *G. inopinata* in the insertion: E.N. Akulov.

Based on extensive pheromone monitoring and the examination of a substantial number of *G. inopinata* specimens (31,962 males) in Russia, we significantly expanded the known range of *G. inopinata* (Figure 8). The species was historically known to be restricted to Eastern Siberia and Far East regions (Russia), northeastern China, and Japan [1]. In 2011, we, for the first time, detected the moth in Krasnoyarsk Krai and Khakassia Republic [22]. Our present findings extended the species’ western range boundary by 35° in longitude, from 91° E (the previously westernmost published record from Khakassia Republic) [22] to approximately 56° E in Perm Krai. As a result, the current distribution of *G. inopinata* spans latitudes 32 to 58° N and longitudes 56 to 143° E (Figure 8), doubling the previously known range of this species.

Notably, we detected the moth in eight administrative regions of Russia: in seven regions in the Asian part of the country (mostly in Western Siberia) and in one in the European part. In the latter case, the moth was revealed in Perm Krai, a region adjacent to the Ural Mountains and located approximately 1100 km west (in a straight line) from the westernmost point in Siberia (Omsk Oblast) documented in this study (Figure 8).

We suggest that the apparent gap in the current distribution of *G. inopinata* (i.e., Sverdlovsk and Tyumen Oblasts; Figure 8) is likely due to a lack of data (as pheromone monitoring was not conducted in these regions), rather than a true absence of the species. It is highly likely that *G. inopinata* is present in these two regions, and its modern range is continuous rather than fragmented.

It remains uncertain whether *G. inopinata* is expanding its range or has historically been present in the newly recorded regions, but remained overlooked. At present, we lack sufficient evidence to answer these questions. However, it is important to emphasize that before our study, *G. inopinata* was not known in Western Siberia [16]. Its presence, however, was suspected in regions bordering Krasnoyarsk Krai and Khakassia Republic, where the moth was detected in 2011, already at a high population density [22,39].

The detection of this East Asian species in European Russia was particularly unexpected. This represents the first confirmed record of *G. inopinata* in Europe, aside from an accidental interception of a single individual in Finland [8], which was not followed by any evidence of its establishment. Notably, in Perm Krai (European Russia), *G. inopinata* was recorded at a moderate density, with approximately 17 males captured per pheromone trap per week and a total of 83 individuals collected over four weeks (starting in the second half of June 2023). We strongly believe that the species is established in this region, as males were consistently trapped in private gardens with apple trees in a village on the outskirts of Perm city. This location is remote from fruit markets and warehouses, where accidental introductions of fruit pests through imported fruits may occur [51]. The continuous presence of the moth in a residential area with host plants suggests that *G. inopinata* has a stable population in Perm Krai.

The northern distribution boundary of *G. inopinata*, particularly in Siberia (Figure 8), should be considered provisional. Further detailed surveys are needed to confirm the northernmost limit of the moth’s distribution.

Although our observations were conducted intermittently over different years and primarily aimed at confirming the presence of *G. inopinata* in various regions of Russia, the data obtained provide valuable insights into the moth’s abundance and seasonality. Since pheromone traps were deployed during June–July, the period when adult flight typically occurs [22,39], our findings give an idea of population density across surveyed regions. In 2021–2024, *G. inopinata* was generally found having low population density in most regions, with fewer than 10 males per trap per week. However, in two regions of Western Siberia (Omsk Oblast and Altai Krai) and in Perm Krai, the species was recorded at moderate densities, exceeding 10 males per trap per week. In 2014–2018, moderate to high population densities were recorded in Krasnoyarsk Krai, with mass occurrences of *G. inopinata* in the south of the region in 2017 and 2018.

Our study provides further evidence of *G. inopinata*’s ability to produce two generations per year, depending on regional climatic conditions. Through weekly monitoring over more than four months (late May to early October) for five consecutive years, we observed that in the central part of Krasnoyarsk Krai, the species had a single peak in male flight activity, indicating a univoltine life cycle. However, in the southern part of the region, two distinct peaks in male flight activity were observed across multiple years, clearly suggesting the presence of the second generation. Prior to our study, only a univoltine life cycle was reported for the Asian part of Russia [15,17].

Our findings are consistent with previous studies from East Asia, particularly northeastern China, where *G. inopinata* has been shown to develop in either one or two generations annually, depending on local temperature conditions [19,23]. These results underscore the role of regional climate in shaping the voltinism patterns of this pest.

For the related species *G. molesta*, it has been demonstrated that temperature fluctuations significantly influence the species’ development, seasonal activity, and overall population dynamics [52,53]. The degree-day approach, which quantifies the accumulated heat units above a species-specific developmental threshold, enhances the precision of pest management by providing a scientific basis for predicting population dynamics [53]. Detailed studies on the developmental thresholds for *G. molesta* have identified the lower threshold of 9.5 °C and the upper threshold around 31 °C, which are used to calculate the degree-days required for the development of a full generation of the pest [52,54].

In contrast, for *G. inopinata,* the developmental thresholds have not yet been studied, making it impossible to accurately define the number of degree-days required for the development of its generation. This represents an important gap in knowledge, and addressing these issues should be a focus of future research.

Understanding the distribution and abundance of *G. inopinata* is critical for improving pest risk assessment and developing early detection methods, particularly in regions where the species is not yet established but may pose a future threat. Our research is particularly timely given the potential of *G. inopinata* to spread into new areas, including those in the European Union, where it could impact apple and other cultivated fruit crops [7]. The use of pheromone traps in combination with degree-day models could optimize pest management. Additionally, the observed variations in voltinism suggest that region-specific approaches may be necessary, as areas supporting two generations per year may require more intensive monitoring and control measures.

## 5. Conclusions

Our study underscores the value of long-term pheromone monitoring in detection, population assessments, and insights into the seasonal activity patterns of the East Asian fruit-boring pest, *G. inopinata*. Although it was not possible to prove that the species is spreading, we provided valuable data on its current range in Russia. The species occurrence in the European part of Russia combined with its capacity for bivoltinism and mass occurrence under favorable conditions pose new challenges for pest management in Russia and beyond, in particular in Europe and other world regions, where the moth is treated as a quarantine pest. Future research should focus on addressing questions concerning the pest range expansion, involving the use of molecular genetic tools to define its distribution features. Moreover, detailed research would be needed to refine predictive models (incorporating climatic variables), assess the effectiveness of pheromone-based control strategies, and explore natural enemies and their potential for controlling *G. inopinata*. By integrating these approaches, it may be possible to develop more sustainable and region-specific management practices to mitigate the potential impact posed by *G. inopinata.*

## Data Availability

The original contributions presented in this study are included in the article/supplementary material. Further inquiries can be directed to the corresponding author.

## Supplementary Materials

The following supporting information can be downloaded at: https://www.mdpi.com/article/10.3390/life15040521/s1, Table S1: The studied localities, dates and the number of used traps for pheromone monitoring of *Grapholita inopinata* in Russia in 2014–2018 and 2021–2024; Table S2: Seasonal flight activity of *Grapholita inopinata* males (A) and mean daily temperature dynamics (B) modeled using a polynomial function for two regions of Krasnoyarsk Krai (2014–2018).
