# Supplementary material for: Western Range Limit, Population Density, and Flight Dynamics of the Fruit Pest Grapholita inopinata (Lepidoptera: Tortricidae) in Russia
Source: Life (Basel). 2025 Mar 22;15(4):521. doi: 10.3390/life15040521 (PMC12029037; doi:10.3390/life15040521)
Supplement: Supplementary file 1 [file life-15-00521-s001.zip › Table S1.pdf]

Western Range Limit, Population Density, and Flight Dynamics of the Fruit Pest *Grapholita inopinata* (Lepidoptera: Tortricidae) in Russia

Evgeny N. Akulov, Margarita G. Kovalenko, Julia A. Lovtsova, Dmitrii L. Musolin, Natalia I. Kirichenko

Table S1. The studied localities, dates and the number of used traps for pheromone monitoring of *Grapholita inopinata* in Russia in 2014–2018 and 2021–2024.

Administrative region[1]	Year	Number of localities[2]	Location name	Garden type	Dates	Total number of traps used[3]	Total number of trapped males[4]
Kemerovo Oblast	2021	2	Kemerovo district, Urmanay vil.	Private garden	17.VI–03.VII	2	6
			Mariinsk district, Mariinsk	Private garden	03–18.VII	2	10
Tomsk Oblast	2021	1	Tomsk district, Belousovo vil.	Private garden	15.VI–04.VII	6	1
Novosibirsk Oblast	2021	2	Novosibirsk district, Krasnoobsk	Private garden	15–25.VI	2	14
			Novosibirsk	Central Siberian Botanical Garden SB RAS	15–25.VI	2	7
Altai Krai	2022	8	Slavgorod	Private garden	18–28.VI	2	61
			Kulunda vil.	Ornamental apple trees in periurban area	18–28.VI and 11–21.VII	4	123
			Zavyalovsky district, Glubokoe vil.	Private garden	11–26.VI	2	39
			Rubtsovsky district, Veseloyarsk settl.	Private garden	11–26.VI	4	1
			Loktevsky district, Ustyanka settl.	Private garden	15–25.VII	2	47
			Loktevsky district, Georgievka settl.	Private garden	15–25.VII	2	74
			Zmeinogorsk district, Zmeinogorsk	Private garden	11–21.VI	2	64
			Barnaul	Private garden	11.VI–25.VII	15	126
Altai Republic	2022	1	Gorno-Altaysk	Private garden	11–26.VI	16	50
Omsk Oblast	2022	1	Omsk	Private garden	10.VI–13.VII	17	293
Perm Krai	2023	5	Kondratovo vil.	Private garden	22.VI–21.VII	3	83
			Stolbovo	Private garden	08.VII–07.VIII	3	0
			Merkushevo vil.	Private garden	22.VI–21.VII	3	0
			Shemeti vil.	Private garden	25.VI–24.VII	3	0
			Gari vil.	Private garden	23.VI–23.VII	3	0
Kirov Oblast	2023	2	Kirov	Private garden	26.VII–27.VII	4	0
			Yuriyanovsky District	Private garden	06–19.VIII	2	0

Administrative region[1]	Year	Number of localities[2]	Location name	Garden type	Dates	Total number of traps used[3]	Total number of trapped males[4]
Orenburg Oblast	2023	1	Orenburg	Private garden	13.VI–04.VII	8	0
Stavropol Krai	2021	1	Pyatigorsk	Private garden	25.V–07.VI	14	0
Irkutsk Oblast	2023	2	Selivanikha settl.	Private garden	17.VI–04.VII	10	48
			Shelekhovsky district	Private garden	17.VI–24.VI	8	44
Udmurtia Republic	2024	1	Votkinsky District, Fetiki	Private garden	01.VI–30.VI	15	0
Krasnoyarsk Krai, center	2014	3	Krasnoyarsk, Vetluzhanka	Private garden	10.VI–23.IX	48	57
			Krasnoyarsk, Studgorodok	Private garden	10.VI–23.IX	48	390
			Krasnoyarsk, Sverdlovsky district	KBG*	10.VI–23.IX	96	267
	2015	3	Krasnoyarsk, Vetluzhanka	Private garden	29.V–01.X	57	131
			Krasnoyarsk, Studgorodok	Private garden	29.V–01.X	57	517
			Krasnoyarsk, Sverdlovsky district	KBG	29.V–01.X	96	845
	2016	3	Krasnoyarsk, Vetluzhanka	Private garden	19.V–28.IX	60	278
			Krasnoyarsk, Studgorodok	Private garden	19.V–28.IX	60	913
			Krasnoyarsk, Sverdlovsky district	KBG	19.V–28.IX	120	1877
	2017	3	Krasnoyarsk, Vetluzhanka	Private garden	11.V–27.IX	63	792
			Krasnoyarsk, Studgorodok	Private garden	11.V–27.IX	63	2230
			Krasnoyarsk, Sverdlovsky district	KBG	11.V–27.IX	126	1477
	2018	3	Krasnoyarsk, Vetluzhanka	Private garden	24.V–26.IX	57	114
			Krasnoyarsk, Studgorodok	Private garden	24.V–26.IX	57	511
			Krasnoyarsk, Sverdlovsky district	KBG	24.V–26.IX	114	1142
Krasnoyarsk Krai, south	2015	1	Minusinsk district, Opytnoe pole settl.	MES**	29.V–17.IX	102	1245
	2016	1	Minusinsk district, Opytnoe pole settl.	MES	19.V–14.IX	162	2455
	2017	1	Minusinsk district, Opytnoe pole settl.	MES	18.V–13.IX	162	8944
	2018	1	Minusinsk district, Opytnoe pole settl.	MES	17.V–12.IX	162	6416

[1]Number of administrative regions studied = 13 (note: in Krasnoyarsk Krai, two territories were surveyed – center and south).
[2]Number of localities involved = 31 (note: number of localities in Krasnoyarsk Krai repeated in 2014–2015).
[3]Total number of traps used = 1866, among which total number of traps in which *G. inopinata* males were revealed = 1811.
[4]Total number of *G. inopinata* males trapped = 31692.
*KBG – V.M. Krutovsky Botanical Garden.
**MES – Minusinsk Experimental Station for Horticulture and Melon Growing of the Krasnoyarsk Research Institute of Agriculture.
