# Supplementary material for: Western Range Limit, Population Density, and Flight Dynamics of the Fruit Pest Grapholita inopinata (Lepidoptera: Tortricidae) in Russia
Source: Life (Basel). 2025 Mar 22;15(4):521. doi: 10.3390/life15040521 (PMC12029037; doi:10.3390/life15040521)
Supplement: Supplementary file 1 [file life-15-00521-s001.zip › Table S2.pdf]

Western Range Limit, Population Density, and Flight Dynamics of the Fruit Pest *Grapholita inopinata* (Lepidoptera: Tortricidae) in Russia

Evgeny N. Akulov, Margarita G. Kovalenko, Julia A. Lovtsova, Dmitrii L. Musolin, Natalia I. Kirichenko

Table S2. Seasonal flight activity of *Grapholita inopinata* males (A) and mean daily temperature dynamics (B) modeled using a polynomial function for two regions of Krasnoyarsk Krai (2014–2018).

A – Approximation of *G. inopinata* flight activity

Year	Equation	R^2	N*	*p*
Central part of the region				
2014	$y = 3E\text{-}09x^6 - 0.0008x^5 + 85x^4 - 5E{+}06x^3 + 1E{+}11x^2 - 2E{+}15x + 2E{+}19$	0.76	15	< 0.05
2015	$y = 1E\text{-}09x^6 - 0.0003x^5 + 31x^4 - 2E{+}06x^3 + 6E{+}10x^2 - 9E{+}14x + 7E{+}18$	0.89	18	< 0.01
2016	$y = 3E\text{-}09x^6 - 0.0008x^5 + 83x^4 - 5E{+}06x^3 + 2E{+}11x^2 - 3E{+}15x + 2E{+}19$	0.82	19	< 0.05
2017	$y = 8E\text{-}09x^6 - 0.002x^5 + 218x^4 - 1E{+}07x^3 + 4E{+}11x^2 - 7E{+}15x + 5E{+}19$	0.78	20	< 0.05
2018	$y = 5E\text{-}09x^6 - 0.0012x^5 + 129x^4 - 7E{+}06x^3 + 2E{+}11x^2 - 4E{+}15x + 3E{+}19$	0.72	18	< 0.05
Southern part of the region				
2015	$y = -8E\text{-}09x^6 + 0.0021x^5 - 216x^4 + 1E{+}07x^3 - 4E{+}11x^2 + 7E{+}15x - 5E{+}19$	0.99	16	< 0.001
2016	$y = 6E\text{-}09x^6 - 0.0016x^5 + 174x^4 - 1E{+}07x^3 + 3E{+}11x^2 - 5E{+}15x + 4E{+}19$	0.81	18	< 0.01
2017	$y = 2E\text{-}08x^6 - 0.0054x^5 + 576x^4 - 3E{+}07x^3 + 1E{+}12x^2 - 2E{+}16x + 1E{+}20$	0.83	17	< 0.01
2018	$y = 1E\text{-}08x^6 - 0.0033x^5 + 354x^4 - 2E{+}07x^3 + 7E{+}11x^2 - 1E{+}16x + 8E{+}19$	0.75	17	< 0.01

B – Approximation of mean daily temperature dynamics

Year	Equation	R^2	N*	*p*
Central part of the region				
2014	$y = -0.003x^2 + 310x - 6E{+}06$	0.67	134	< 0.05
2015	$y = -0.003x^2 + 254x - 5E{+}06$	0.73	134	< 0.01
2016	$y = -0.003x^2 + 217x - 5E{+}06$	0.66	134	< 0.05
2017	$y = -0.002x^2 + 206x - 4E{+}06$	0.70	134	< 0.01
2018	$y = -0.002x^2 + 162x - 4E{+}06$	0.45	134	< 0.05
Southern part of the region				
2015	$y = -0.003x^2 + 232x - 5E{+}06$	0.63	134	< 0.05
2016	$y = -0.002x^2 + 0.26x + 14$	0.67	134	< 0.05
2017	$y = -0.002x^2 + 0.24x + 15$	0.70	134	< 0.01
2018	$y = -0.002x^2 + 180x - 4E{+}06$	0.53	134	< 0.05

N – number of observations
